# Organisation

**Published:** 1892-07-02

**Authors:** 


### The Institutional Workshop.

### HOSPITAL ADMINISTRATION.

### I.—ORGANISATION.

The term "hospital administration" is usually intended to comprise all the necessary arrangements and management of every department of hospital work except such as exclusively concern the purely medical treatment of the sick. Even here, it is not easy to draw a distinction between the medical and administrative arrangements, since the medical officers employed to minister to the requirements of the sick have expressed duties assigned them by the central administration, and are responsible to it for their due performance. For convenience in keeping accounts, subdivision of labour, and for purposes of comparison, it is usual in some hospitals to draw a kind of arbitrary distinction between maintenance and administration, but as the former is comprised in the management as much as is the maintenance of the building and its environs, it is hardly possible to dissociate one aspect of the question from the other. In most countries where the hospitals are largely dependent on voluntary help, and in Great Britain and Ireland, where, with few exceptions they are entirely dependent on it, the onus of management lies directly with the contributors who select certain representatives of their number to manage the affairs of the charity. These again form themselves into committees according with the special requirements, as house, finance, medical, nursing, &c., and are usually presided over by a Chairman familiar with the work of the establishment. As the business is entirely of a voluntary character, it rarely happens, except on special occasions, that meetings of committees are fully attended, and, as a natural consequence, the supervision of accounts and the various domestic requirements of the charity are relegated to the Chairman and a few directors best acquainted with the needs of the establishment. There can be no doubt about the distinct advantage of this arrangement, seeing that the perfunctory and irregular attendance of a larger number would not tend to expedite business matters. The most important committee is that which in most hospitals has to meet weekly, and is usually named the House Committee. To it the chief officers of the establishment have to submit their reports and to take instructions, while the committee has to pass bills, sanction requirements, and to discuss matters bearing on the well-being of the charity. In large hospitals rules and bye-laws are in force for the guidance of the various officials, which are altered, modified, or added to from time to time to meet recurrent requirements, and in order that these should be strictly complied with, there is, or should be, in every hospital a resident officer to act in the absence of the Visiting Committee in all cases of doubt or difficulty there may arise, and to carry on the necessary correspondence. In England, for the most part, this duty falls to the lot of a lay Secretary or Superintendent, but in Scotland, Ireland, America, and in most English-speaking countries it is performed by a resident medical man, while in small hospitals and poor law infirmaries it devolves on the House Surgeon and Medical Superintendent respectively.

It rests with the Committee of Management in all cases to receive periodically, either by open advertisement or wise selection, tenders for the supply of the main articles of consumption, such as coals, meat, bread, milk, &c. Their duty is also to make the best arrangement they can through accredited wholesale or retail houses, for supplies of drugs, and especially surgical dressings, a source of expenditure which has largely increased during the last twenty years, especially in such hospitals as are associated with medical schools. In these, which now exist in all large cities and University towns, it is necessary, in the interests of education and research, that there should be a much larger visiting staff than would be considered necessary in a county infirmary of equal dimensions, and there is every probability as time advances that there will be a progressive increase to the number for the treatment of the out as well as of the in-patients in general hospitals. The same remark applies to the resident medical or subordinate staff of officers in more immediate charge of the sick, qualified men selected from the more distinguished students, and whose terms of office are terminable within a year or six months, and even in shorter periods. The important question of sanitation in hospitals is one requiring incessant vigilance, and has hitherto been left almost entirely to surveyors called in on pressing emergencies, but there is good reason for believing that in the future it will become more and more the duty of experts on Committees of Management to supervise these matters. Very often they are referred to a mixed commission of the governors and medical staff of the hospital, and it is always advantageous to obtain the opinions of those most intimately concerned in the successful treatment of the sick, though they may not be members of the board. In fact, it is a disputed question whether it would not be to the advantage of the administration that some selected members of the acting staff should be placed on the Committee of Management, but the general opinion appears to favour the opposite practice, possibly on account of the conflicting interests involved in the other.

No subject connected with hospital work has of late years engaged so much attention both on the part of the general public and of hospital authorities as the nursing of the sick, which is undoubtedly a part, and a very important one of hospital administration. Here we have a species of work eminently requiring female supervision of a skilled and experienced character, which must be left in great measure to the judgment and forethought of the lady who fills the office of Matron, and under whose guidance a suitable selection of candidates for probation is made, together with the rules relative to their duties. Most large hospitals, and more especially such as are connected with medical schools, are also training schools for nurses, who, after a period of two or three years, obtain their certificates, and pass out into the world as hospital-trained, much as a student does who has finished his medical curriculum. The effect of the system is a constant change in the personnel of the nurses to make room for new applicants to fill the places of those whose period of probation has expired. There is no special inducement except in a very few instances, for the better class of woman to continue long in the service of the hospitals after they have had a full insight into their work, as their services are eagerly sought after, either for private nursing or to fill superior posts in kindred institutions where they are better remunerated. The organization of the nursing arrangements must necessarily in the first instance fall to the lot of the Committee of Management, but its practical working must be left largely in the hands of the Matron, aided by the resident officer. It is but fair, however, that in the event of any one, whether male or female, having a cause of grievance, they should have the power of appeal to the weekly Committee, and this rule is carried out in every well-conducted hospital. To carry on the nursing effectively, it has been found essentially necessary, besides increasing the number of nurse probationers, to add greatly to the number of women employed in the domestic service of the different institutions, as ward maids and scrubbers. The duties of these are sometimes so important as to require the undivided attention of an Assistant Matron, but in small hospitals they can be very well supervised by the Matron. Either one or more Night Superintendents of nurses are now employed in all large hospitals to look after the due performance of their duties by the night nurses, attend operations or serious casualties that may occur during the night, and otherwise to make themselves useful. These offices are very important, and should only be filled by persons of experience and ability, and on account of the irksome nature of the duties it is desirable that they should not be held too long by the same individuals. In cottage and small hospitals containing from ten to fifty beds, the Matron or Sister in charge, besides superintending the nursing arrangements has also the custody of the linen store, and has to act as general housekeeper, while in larger hospitals persons are specially appointed to these offices who need have no knowledge of nursing, but who, like all females in the establishment, should be immediately under the control of the Matron. It has been found absolutely necessary to the proper and harmonious working of all departments of hospitals that there should be perfect unity of purpose among the several officials engaged in carrying out the wishes of the central authority, and to maintain this intact, full confidence and discretion must be placed in the motives of those officers which the Committee allows the necessary disciplinary powers.

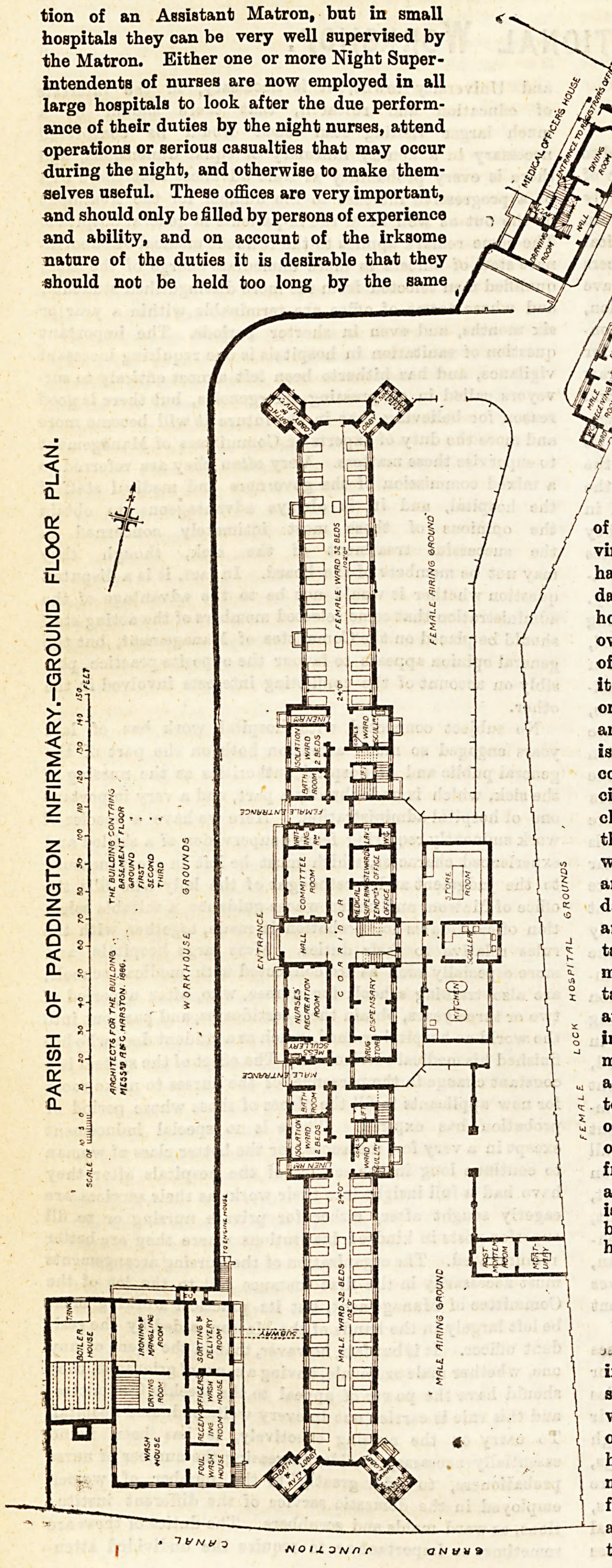

PARISH OF PADDINGTON INFIRMARY.—GROUND FLOOR PLAN.

The health of the hospital itself is unquestionably a matter of first importance, and as such falls directly under the province of administration. Though a knowledge of sanitation has become very general among the educated classes, and is daily becoming more diffused, it is doubtful whether a hospital committee are the best judges of what concerns their own interests in maintaining a sound and healthy condition of the buildings they are called on to manage. Seeing this, it has been customary to relegate the duty to an expert or surveyor, who is called in from time to time when anything goes wrong with the drainage, or when it is considered advisable to remodel, or originate any sanitary contrivances tending to the health of the inmates. Under all circumstances such an alternative is necessary, but it is clearly the duty of the committee to have a resident officer on the premises, conversant with the sanitary arrangements, and with the ventilation and warming of the wards and their annexes, and whose duty it should be to visit the wards daily, and to remedy or report on all matters requiring amendment. The vast changes which of late years have taken place in most hospitals, with the view of reducing the mischief consequent on the spread of septic disease, necessitate the utmost care and precaution in dealing with the sick, and though topical application in the form of surgical dressings are exclusively the province of those who have the more immediate charge, it is no less the business of the administration to aid in every way in their power the protective measures, by enforcing cleanliness, and by using other sanitary precautions known to have the best influence on treatment. Buildings erected in pre-sanitary times labour from their construction under many disadvantages, especially as regards freedom of ventilation, and suitable means for isolation, though much has been done to remedy these evils, both by legislation and by the local authority in charge of the hospital.

